# Validity of a computer-assisted manual segmentation software to quantify wrist erosion volume using computed tomography scans in rheumatoid arthritis

**DOI:** 10.1186/1471-2474-14-265

**Published:** 2013-09-12

**Authors:** Fausto Salaffi, Marina Carotti, Alessandro Ciapetti, Alarico Ariani, Stefania Gasparini, Walter Grassi

**Affiliations:** 1 Rheumatology Department, Politechnic University of the Marche, Ospedale “C. Urbani”, Via dei Colli, 52, Ancona, Jesi 60035, Italy; 2 Radiology Department, Politechnic University of the Marche, Ancona, Italy

**Keywords:** Rheumatoid arthritis, Bone erosion, Computed tomography, Conventional radiography, Computer-assisted manual segmentation technique

## Abstract

**Background:**

To investigate the performance of conventional radiography (CR) for the detection of bone erosions of wrist in rheumatoid arthritis (RA) using multidetector computed tomography (CT) as the reference method and to evaluate the validity of a computer-assisted manual segmentation (outlining) technique to quantify erosion volume on CT scans.

**Methods:**

Twenty five RA patients and six controls underwent CT and radiographic evaluation of the dominant wrist on the same day. CT was performed by using a 64 GE light Speed VCT power. Wrists images were evaluated separately and scored for the presence of erosions according to the Outcome Measures in Rheumatology Rheumatoid Arthritis MRI Scoring System (RAMRIS) and the Sharp/van der Heijde scoring method. Measurements of bone erosion volumes were obtained using OsiriX medical imaging software. The mean value of the volumes of the CT bone erosions detected at two readings was used to calculate inter-rater agreement.

**Results:**

The overall sensitivity, specificity and accuracy of radiography for detecting erosions were 25.5%, 98.3% and 70.1%, respectively. Using computer-assisted manual segmentation (outlining) technique, erosion volume on CT measurements per subject was ranged from 0.001 cm^3^ to 2.01 cm^3^. Spearman’s RAMRIS score of each wrist bones in all subjects (n = 25) were correlated with the total erosion volume on CT (p < 0.0001), with the ratio between erosion volume and the corresponding bone volume on a percentage basis (p < 0.0001). The total Sharp/van der Heijde erosion score of the all wrist bones was correlate with the RAMRIS score (p = 0.008). The intraclass correlation coefficients (ICC) for manual segmentation showed high agreement (ICC = 0.901).

**Conclusions:**

Considering CT as the reference method, CR showed very low sensitivity. A close correlation with CT erosion volumes supports the OMERACT RAMRIS erosion score as a semiquantitative measure of joint damage in RA. Although the computer-assisted manual segmentation can be beneficial for diagnostic decision in cross-sectional CT examinations of the wrist in RA, this technique will require further evaluation in terms of responsiveness.

## Background

The optimization of imaging measures is an important strategy for evaluating and monitoring bone damage in rheumatoid arthritis (RA) [1]. Although conventional radiography (CR) remains the cornerstone of imaging modalities in RA, computed tomography (CT) appear more sensitive for the detection of bone erosions in comparison to CR, magnetic resonance imaging (MRI) and ultrasonography (US). For that reason, CT can be considered the standard reference method for the detection of erosive bone destructions in early stage of the disease [2-6]. Current generation of ultrafast CTs allows acquiring high-resolution volumetric data in few seconds and providing detailed anatomical informations. In particular, 3-D volume rendering techniques makes feasible to generate high-quality images, offering a realistic anatomical view of the body and organs from tomographic data.

Radiographic scores, such as the Larsen [7], Sharp scores and its modified versions [8,9], are standard methods to evaluate and monitor the joint damage in RA [10,11]. Despite considerable effort to either reduce or at least define the intrinsic limitations of radiographic scores, some problems remains, especially for the wrist’s evaluation, such as reader variability, floor and ceiling effects [12-14] and inability to accurately quantify damage and its progression. To circumvent these problems, the feasibility of a computerized image analysis of erosion volumes of the hands has been evaluated. The computer system has been shown to provide reproducible data, but showed poor correlation with common standard scores used to define bone erosions [15-18]. Unlike the CR, no method of qualitative score was developed specifically for CT images. Therefore, monitoring changes of the erosions volume is an important task for a correct evaluation of disease progression and response to treatment. Measurements of erosions volume by CT images has been described by Duryea et al. [19] and Døhn et al. [20]. In particular, Duryea et al. [19] have developed a semi-automated method for the evaluation of the profile of carpal bones and subsequent determination of the volume erosion by comparing the images obtained at baseline and subsequent checks.

The objectives of our study were to investigate the performance of the CR for the detection of bone erosions in the wrist of patients with RA, using multidetector CT as the reference method and to assess the validity of a computer-assisted manual segmentation software to quantify the erosion volume of wrist detected by CT.

## Methods

### Patients

Twenty five patients with RA (6 men and 19 women) with a median age of 54 years old (range 35–79 years old) and a mean disease duration of 18 months (range 11–24 months), fulfilling the American College of Rheumatology (ACR) 1987 criteria [21] and six healthy controls (volunteer medical doctor and nurses of the Radiology Department of the Politechnic University of Marche, Ancona, Italy) matched for age and gender, were included in the study. The study was approved by the local Institutional Research Ethics Committee (Comitato Etico dell’Azienda Sanitaria Unica Regionale di Ancona) and informed consent was obtained from all patients.

### Imaging

**Figure 1:**
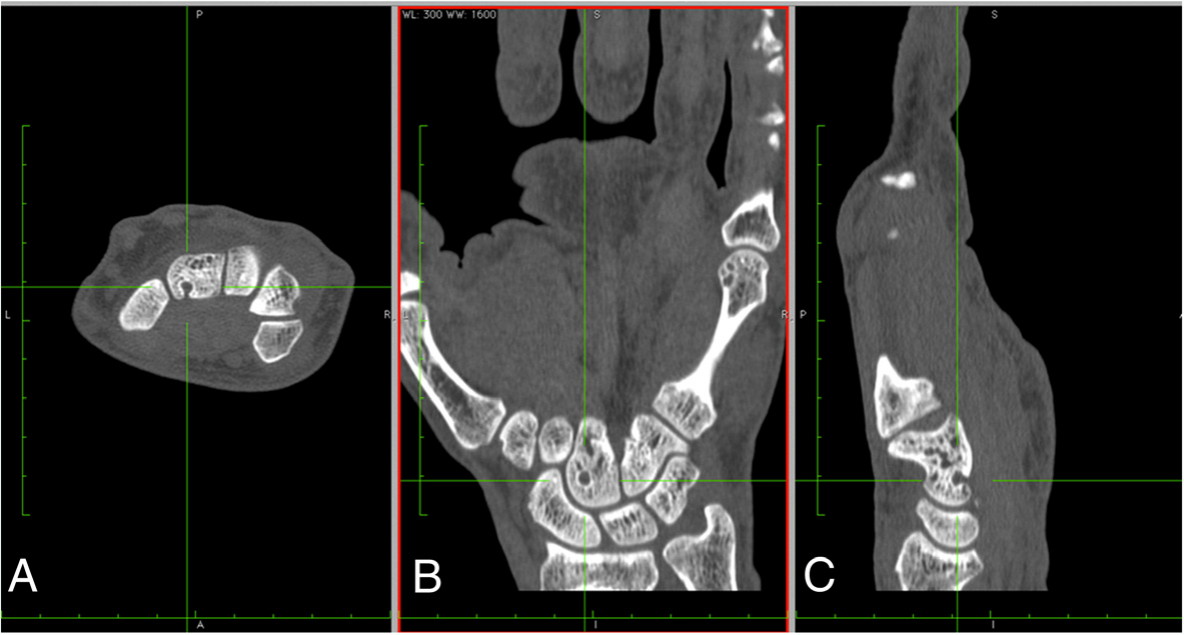
An example of bone erosion of the capitate bone seen on axial (A), coronal (B) and sagittal (C) CT reconstructions of wrist.

The CR was performed at level of the dominant wrist in RA patients and healthy subjects, according postero-anterior and oblique projections. CT images were obtained in the same day by using a 64 GE light Speed VCT power (parameters: 90 kV, 80 mAs, pitch 0.531 mm, slice thickness of 0.625 mm, slice spacing 0.4 mm, gantry rotation time of 0.8 s, without contrast medium). Patients were placed in a prone position with the arm stretched and the palm facing down for the CT examinations. Axial, coronal and sagittal reconstructions, with a slice thickness of 1.0 mm, were created and used for images evaluation (Figure 1).

### Erosion score assessment

Images obtained by CR and CT were evaluated for erosions, by a musculoskeletal radiologist (MC) experienced reader of the Outcome Measures in Rheumatology Rheumatoid Arthritis MRI Scoring (OMERACT RAMRIS) system, blinded to clinical and other imaging data. The presence and number of erosions of the dominant wrist were evaluated by CR and CT examining the following bones: scaphoid, lunate, triquetrum, pisisorm, trapezium, trapezoid, capitate, hamate, distal ulna, distal radius and metacarpal bases. A total of 375 bones were explored. The correct position of erosions was marked on scoring sheets. According to OMERACT criteria [22], erosions on CT were defined as a definite cortical break seen in two planes, with a cortical break (loss of cortex) seen in at least one plane. All three orthogonal planes were viewed to confirm the presence of erosion (Figure 1). Definitions and scorings of CT erosions were described by using the semiquantitative OMERACT RAMRIS [23,24]. The score for erosions were assigned considering the percentage of bone volume involved (score 0–10, by 10% volume increments), leading to a total score ranging from 0 to 150 [23,25]. Erosions of dominant wrist evaluated by CR were scored by Sharp/van der Heijde method [9]. According this method the total erosion score of one wrist ranges from 0 to 75.

### Erosion volume measurements

**Figure 2:**
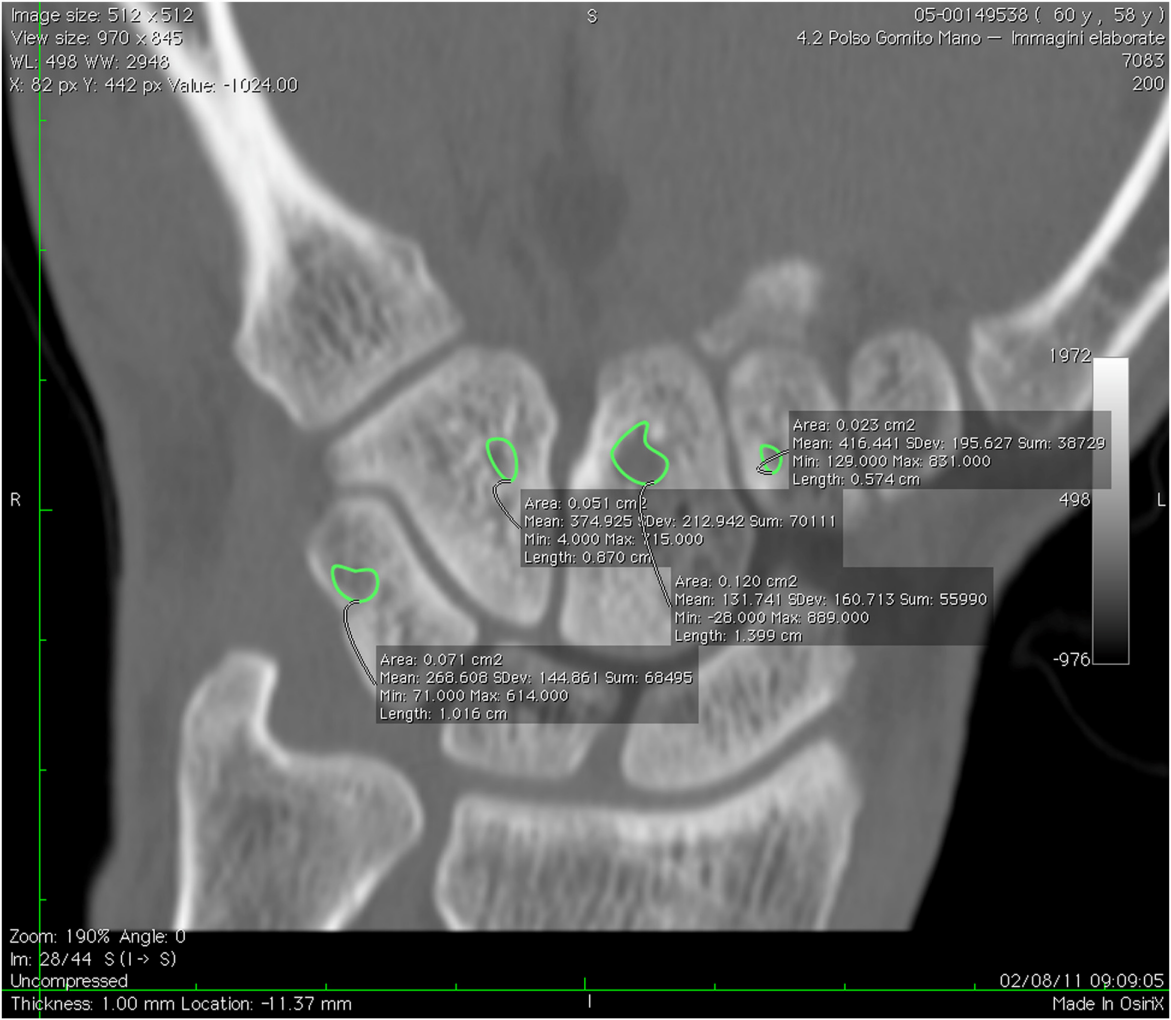
**CT coronal reconstructions of wrist.** Magnification of erosions of triquetrum, hamate, capitate and trapezoid. Each area was automatically obtained by defining the contour of the erosion.

**Figure 3:**
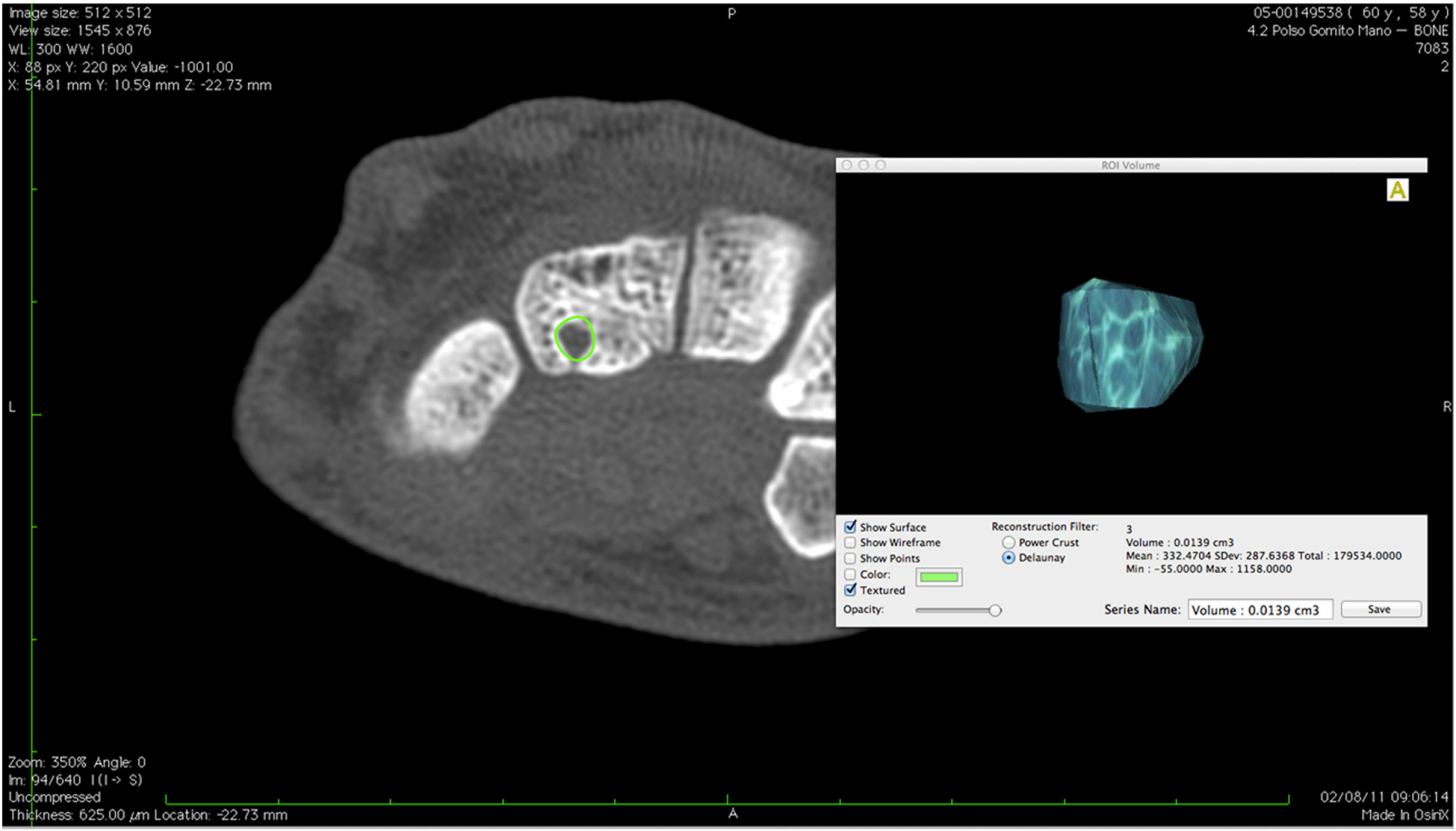
**CT images of wrist *.*** The erosion volume was obtained after the automatic calculation of the area of the erosion.

CT images were read by two rheumatologists (FS and AA) over four days by using a large-screen (27-inch) radiologic workstation monitor. Erosions volumes were calculated by a computer-assisted manual segmentation (outlining) technique using *OsiriX* medical imaging software which is a fast DICOM viewer program for the Apple Macintosh, downloadable at the following Web site: http://www.osirix-viewer.com. The *OsiriX* program offers all the basic image manipulation functions of zoom, intensity adjustment and filtering with real-time performance [26]. Additional functions are accessible as well, such as multiplanar projections, convolution filters, variable slice thickness adjustments, minimum and maximum intensity projections, surface and volume rendering and number of slices to be reconstructed. Each erosion was outlined manually in coronal or axial slice. The outlining of erosion borders was done using a Bamboo Connect pen tablet system (Wacom Technology Corporation, Vancouver, WA, USA). The erosion area was then calculated by the computer software from multiple slices and multiplied by the slice thickness to provide the erosion volume. Further, the ratio between erosion volume (*eV*) and the corresponding bone volume (*bV*), on a percentage basis $\left(e,\overset{\sim }{V},\text{bV},*,100\right)$ have been calculated as well. The mean of the two measurements were used for the analysis. The readers recorded the time consumed scoring each image set. The complete scoring of images from one patient took an average of 18 minutes (ranging from 13 to 48 minutes). Figure 2 give example of manual segmentation of the wrist erosions obtained in coronal projection. Figure 3 shows the calculation of volume erosion of the capitate bone seen in axial scan.

### Statistical analysis

The specificity, sensitivity and accuracy of CR in detecting bone erosions, considering CT as the reference method, were calculated. Spearman’s correlation coefficients between the OMERACT RAMRIS erosion scores, CT erosion volume and the radiographic Sharp/van der Heijde erosion score were obtained. Inter-rater reproducibility was calculated as the intraclass correlation coefficient (ICC) with absolute agreement followed by the 95% confidence intervals (95% CI) in brackets. The mean value of the CT volumes found by two readers was used to calculate the inter-rater agreement. A Bland–Altman difference plot [27] was performed to compare the manual volume measurements. Wilcoxon Signed-Ranks Test was used to investigate significant differences between observers. MedCalc, version 12.0 for Windows (MedCalc Software, Ostend, Belgium) was used for statistical calculations and p value less than 0.05 was considered statistically significant.

## Results

**Figure 4:**
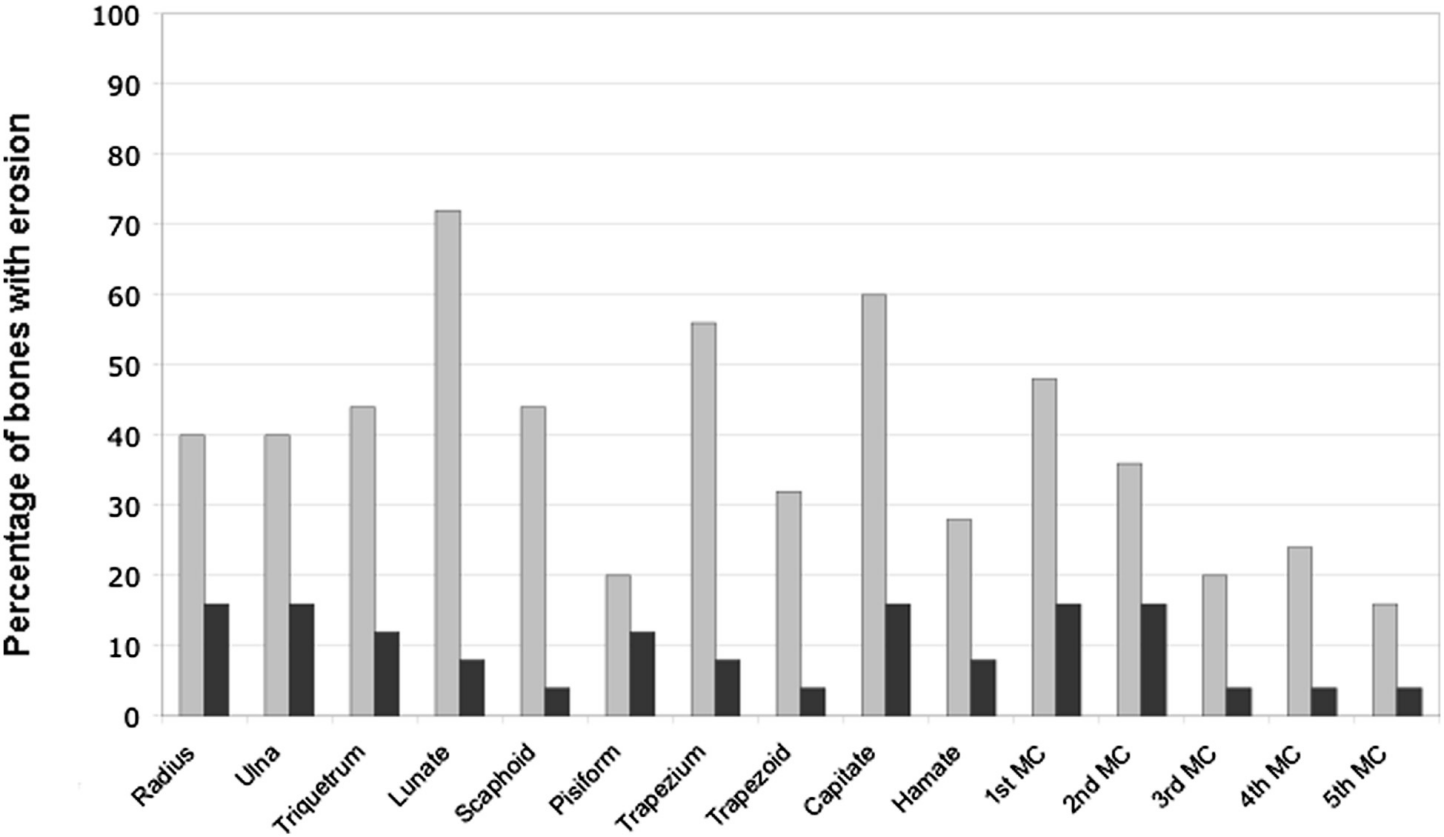
**Proportion of bones (n = 375) with erosions.** Percentage of bones with erosions detected by CT (grey boxes) and CR (black boxes).

A total of 375 wrist bones were assessed for erosions in RA patients and 90 wrist bones in healthy controls. All patients had at least one erosion on CT, while none was seen in healthy controls. CT and CR detected 145 and 37 erosions, respectively. All erosions detected by CR were confirmed by CT. The lunate, trapezium, capitate and the first metacarpal head bones were common sites of erosions (Figure 4). Considering CT as the reference method, the sensitivity, specificity and overall accuracy of CR for detecting erosions were 25.5%, 98.3% and 70.1%, respectively. Pattern differences between early and established disease were not detected.

**Figure 5:**
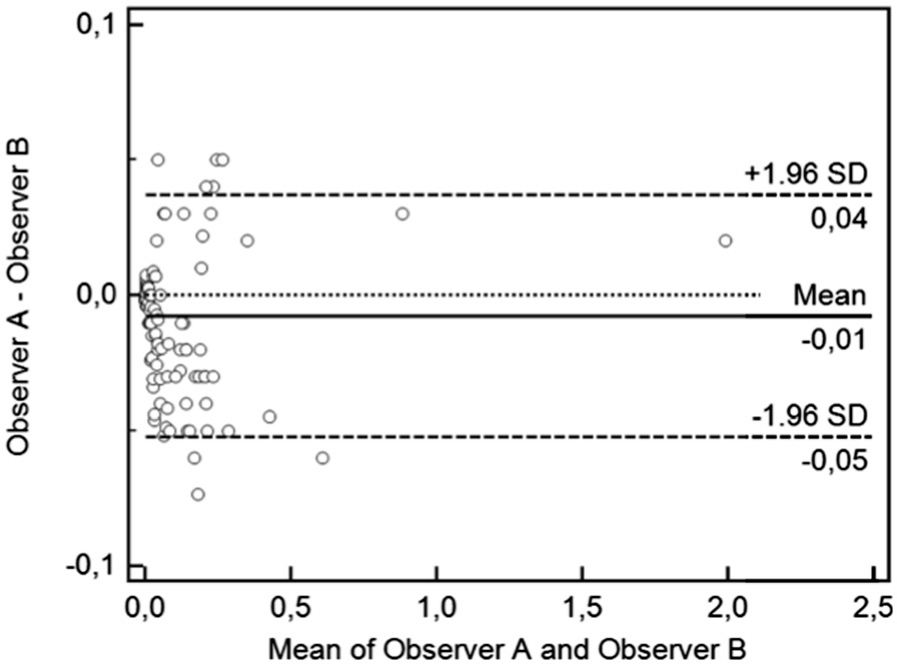
A Bland-Altman difference plot comparing computer-assisted erosion volume measurements by two observers.

The total erosion volume in each subject ranged from 0.001 cm^3^ to 2.01 cm^3^. The highest erosion volumes were noted in the capitate (highest value 0.42 cm^3^) and lunate (highest value 0.26 cm^3^) bones. Spearman’s RAMRIS CT score of individual wrist bones in all patients, correlated with the erosion volume on CT (*rho* 0.823; p < 0.0001) and with the ratio between erosion volume and the corresponding bone volume on a percentage basis (*rho* = 0.914; p < 0.0001). The total Sharp/van der Heijde erosion score of the all wrist bones was slight correlated with the RAMRIS CT score (*rho* 0.143; p = 0.008), but not with total volume erosion measurement on CT (*rho* 0.098; *P* = 0.069). The ICC for manual segmentation showed very high precision (ICC = 0.901; 95% CI 0.882 to 0.931), with no significant difference in the inter-rater reproducibility. The mean and 95% CI of the absolute difference between the volume measurements were 0.158 (95% CI for the mean 0.105 to 0.211), and 0.165 (95% CI for the mean 0.1081 to 0.222) cm^3^, respectively (difference 0.00924; 95% of difference −0.069 to 0.088), which are depicted graphically in the Bland–Altman difference plot of Figure 5.

## Discussion

Considering the wrist frequently involved in RA [28,29] and that anatomical damage at this level has a predictive significance of evolution of the disease, its evaluation assumes an important clinical significance [28-32]. The lunate, trapezium, capitate and the first metacarpal head bones are common sites of erosions. Østergaard et al [33] found that capitate, ulna, lunate, triquetrum, and scaphoid bones were the most frequently bone involved in a MRI datasets from 258 RA patients (126 early RA patients).

Experiences from comparative studies of CR, US, MRI and CT have shown that CT is the most sensitive imagine technique for the detection of bone erosions [2,3,34-38] and, therefore, can be considered the gold standard for the evaluation of wrist anatomical damage in RA. Difficulties of CR for a detail evaluation of wrist joints are mainly due to a projectional superimposition of bones in this complicated anatomical area, high irregularities of the bone margins (e.g. at level of ligaments attachment) and the presence of nutritive foramina that can appear like erosions. These aspects make difficult the discrimination between normal anatomy and presence of erosions.

Measurements of erosion progression in patients with RA have generally relied upon semiquantitative scoring systems [7,11,14]. Despite considerable effort to either reduce or at least define the intrinsic limitations of radiographic scores, problems remain with reader variability and floor and ceiling effects [12-14]. Principles of OMERACT RA MRI system [23,24] were applied to score bone erosions by CT. Despite a scoring system for CT images is not yet available, we found OMERACT RA MRI system reasonable to use due to the similar tomographic visualization of joints on CT and MRI, the good inter-intraobserver reliability and a high level of sensitivity and specificity demonstrated by RAMRIS system in monitoring joint destruction in RA [39-41].

Recently, technological advances in CT imaging modalities and the introduction of 3D image reconstruction through computer systems, allowed the visualization of tiny defects of the wrist a feasible task [42]. High resolution CT systems are fast and produce high spatial resolution images with near isotropic voxels. Therefore, 3D image reconstruction of segmented carpals is significantly more accurate. The visualization and processing of 3D data require special navigation tools and multidimensional rendering software that are available on high end 3D rendering workstations that are most accessible in academic and specialized imaging centers.

The successful diffusion of *OsiriX* in the medical community [43] is mainly due to the fact that it is a cost effective alternative to not-free softwares and can be customized to match the needs and specific usage in clinical setups [26]. Previously published studies have investigated segmentation of the carpal bones of wrist by CT scans with a variety of goals. Snel et al. [44,45] and Sebastian et al. [46] performed wrist segmentation to examine the kinematics of the carpal bones. Other studies have examined wrist scans as a validation of the general 3D segmentation methods. Van Cleynenbreugel et al. [47] and Yao et al. [48] described a semiautomatic method to segment bones on spiral CT scans. These techniques were developed to better understand carpal kinematics or as part of a more general 3D segmentation package. As the same Døhn et al. [49] and Bird et al. [50], we found that the volume measurements of erosions by CT was highly reproducible and closely correlated with the semiquantitative scores of bone erosions according to OMERACT RAMRIS system score. This aspect support the evidence for the semiquantitative MRI measures of erosion as a valid measure of bone destruction in RA. Although the volume measurement of erosion by CT has proved to yield reproducible findings, it showed poor correlation with joint space narrowing score [15] and with standard erosion score [18]. Since CT scanning appears more accurate than the observation of 2D images, this result can compromise the validity of the total Sharp/van der Heijde erosion score. However, the disadvantages of CT scanning are due to its relatively higher cost and risk of hazard from radiation exposure [51,52]. Further, our method evaluates 15 carpal sites in one shot, each of them potentially containing multiple erosions. A limited number of joints can be examined by using CT due to the exposure to ionizing radiation. Consequently, a smaller number of sites can be assessed by CT which potentially lead to a risk of missing signs of erosive progression of the disease. Additionally, the method could also be developed to measure erosion volumes in the proximal interphalangeal and metacarpophalangeal joints that are common sites of erosions as well.

These considerations could account for the poor correlation observed between the total Sharp/van der Heijde erosion score and the total erosion volume measurement on CT. This might reside in the fact that the first is a semiquantitative measure of the erosion based on the simple observation of two-dimensional images, while *OsiriX* allows to measure real volumes.

## Conclusions

The number of erosions detected on CT indicates that this imaging modality is high sensitive for detecting bone erosions in wrist of RA patients. The close correlation with erosion volumes determined by CT provides further evidence for the semiquantitative OMERACT RAMRIS erosion score as a valid tool of anatomical bone damage evaluation. However, the sensitivity to change is not yet established and the disadvantages of CT include the necessity for ionising radiation and inability to visualize soft tissue. Moreover, the computer-assisted manual segmentation technique is complicated and time-consuming. The longer time for the volume calculations was largely due to the manual nature of the segmentation process as well as to the large number of bones assessed at level of wrist. Whether computer-assisted manual segmentation technique is sufficiently precise the evaluation of responsiveness has not yet been tested in our study and, therefore, it will be necessary to evaluate this aspect in further investigations on larger prospective studies.

## Abbreviations

RA: Rheumatoid arthritis; CR: Conventional radiography; CT: Computed tomography; MRI: Magnetic resonance imaging; US: Ultrasonography; ACR: American College of Rheumatology (ACR); OMERACT RAMRIS: Outcome measures in rheumatology rheumatoid arthritis MRI scoring system; ICC: Intraclass correlation coefficient.

## Competing interests

The authors declare that they have no competing interests.

## Authors’ contributions

**FS** contributed to the conception and design of the study, to perform the data analysis and was in charge of drafting and writing the manuscript. **MC** contributed to the conception and design of the study, to the acquisition of data and in writing the manuscript. **AC** contributed in writing the manuscript. **AA** contributed to acquisition of data; **SG** contributed to the acquisition of data. **WG** contributed in writing the manuscript. All the authors read and approved the final manuscript.

## Pre-publication history

The pre-publication history for this paper can be accessed here:

http://www.biomedcentral.com/1471-2474/14/265/prepub
